# Peptic Ulcer Disease in Healthcare Workers: A Nationwide Population-Based Cohort Study

**DOI:** 10.1371/journal.pone.0135456

**Published:** 2015-08-24

**Authors:** Hong-Yue Lin, Shih-Feng Weng, Hung-Jung Lin, Chien-Chin Hsu, Jhi-Joung Wang, Shih-Bin Su, How-Ran Guo, Chien-Cheng Huang

**Affiliations:** 1 Department of Emergency Medicine, Chi-Mei Medical Center, Tainan, Taiwan; 2 Departments of Medical Research, Chi-Mei Medical Center, Tainan, Taiwan; 3 Department of Healthcare Administration and Medical Informatics, Kaohsiung Medical University, Kaohsiung, Taiwan; 4 Department of Biotechnology, Southern Taiwan University of Science and Technology, Tainan, Taiwan; 5 Department of Emergency Medicine, Taipei Medical University, Taipei, Taiwan; 6 Department of Leisure, Recreation and Tourism Management, Southern Taiwan University of Science and Technology, Tainan, Taiwan; 7 Department of Occupational Medicine, Chi-Mei Medical Center, Tainan, Taiwan; 8 Department of Medical Research, Chi Mei Medical Center, Liouying, Tainan, Taiwan; 9 Department of Environmental and Occupational Health, College of Medicine, National Cheng Kung University, Tainan, Taiwan; 10 Department of Occupational and Environmental Medicine, National Cheng Kung University Hospital, Tainan, Taiwan; 11 Department of Child Care and Education, Southern Taiwan University of Science and Technology, Tainan, Taiwan; 12 Department of Geriatrics and Gerontology, Chi-Mei Medical Center, Tainan, Taiwan; University Hospital Llandough, UNITED KINGDOM

## Abstract

Health care workers (HCWs) in Taiwan have heavy, stressful workloads, are on-call, and have rotating nightshifts, all of which might contribute to peptic ulcer disease (PUD). We wanted to evaluate the PUD risk in HCWs, which is not clear. Using Taiwan’s National Health Insurance Research Database, we identified 50,226 physicians, 122,357 nurses, 20,677 pharmacists, and 25,059 other HCWs (dieticians, technicians, rehabilitation therapists, and social workers) as the study cohort, and randomly selected an identical number of non-HCW patients (i.e., general population) as the comparison cohort. Conditional logistical regression analysis was used to compare the PUD risk between them. Subgroup analysis for physician specialties was also done. Nurses and other HCWs had a significantly higher PUD risk than did the general population (odds ratio [OR]: 1.477; 95% confidence interval [CI]: 1.433–1.521 and OR: 1.328; 95% CI: 1.245–1.418, respectively); pharmacists had a lower risk (OR: 0.884; 95% CI: 0.828–0.945); physicians had a nonsignificantly different risk (OR: 1.029; 95% CI: 0.987–1.072). In the physician specialty subgroup analysis, internal medicine, surgery, Ob/Gyn, and family medicine specialists had a higher PUD risk than other physicians (OR: 1.579; 95% CI: 1.441–1.731, OR: 1.734; 95% CI: 1.565–1.922, OR: 1.336; 95% CI: 1.151–1.550, and OR: 1.615; 95% CI: 1.425–1.831, respectively). In contrast, emergency physicians had a lower risk (OR: 0.544; 95% CI: 0.359–0.822). Heavy workloads, long working hours, workplace stress, rotating nightshifts, and coping skills may explain our epidemiological findings of higher risks for PUD in some HCWs, which might help us improve our health policies for HCWs.

## Introduction

Peptic ulcer disease (PUD) (gastric and duodenal ulcers) is caused by a disruption of the balance between hostile factors such as *Helicobacter pylori*, gastric acid, nonsteroidal anti-inflammatory drugs (NSAIDs), and pepsin, and protective factors such as mucus, prostaglandins, bicarbonate, and blood flow to the mucosa [1]. The association between PUD and occupation revealed substantial variations in the incidence of different occupations [2]. Previous studies showed that jobs with high stress and responsibility, and shift work contributed to PUD [2]. There is a general agreement that the higher the stress of the occupation, the higher the ulcer rate [2]. This general agreement can be observed in developing countries during the process of industrialization and modernization, PUD is increasing due to the influence of strain and stress [2].

Taiwan has a national health insurance (NHI) program that covers nearly 100% of its legal residents [3]. The NHI provides all enrollees cheap and equal access to healthcare; therefore, it has greatly stimulated the demand for healthcare. After the 1995 launch of the NHI, Taiwan’s healthcare workers (HCWs) (physicians, nurses, pharmacists, dieticians, technicians, rehabilitation therapists, social workers, etc.) might have greater workloads than do HCWs in other nations [4]. For example, the annual number of outpatient visits per physician in Taiwan increased from 6,621 in 1992 to 8,600 in 2012 (+30%). The number of outpatient visits per person was 7.89 in 1992 and increased to 15.2 in 2010 (+93%) [5,6]. Stress and shift work were risk factors for PUD [2,7]; however, there is no study on PUD in HCWs in the literature. Therefore, we conceived this nationwide population-based retrospective cohort study to investigate PUD in HCWs versus the general population. We hypothesized that Taiwan’s HCWs, especially those with more shift work and physicians specialized in emergency and critical care medicine, have a higher risk for PUD than does the general population.

## Methods

### Data sources

Our data are from the Taiwan National Health Insurance Research Database (NHIRD), which contains deidentified patient information [8]. The NHIRD contains claims based on ICD-9-CM (International Classification of Diseases, Ninth Revision, Clinical Modification) codes for clinical diagnoses, prescribed drugs, procedures, dates of outpatient visits, admission and discharge, gender, date of birth, and monthly income [8]. Information specifically on HCWs, including date licensed, specialty, work area, types of hospitals worked in, types of employment, and encrypted identification number, is also available in the Registry of Medical Personnel. The information can be linked to the NHIRD. All the expenses for PUD, hypertension (HTN), diabetes mellitus (DM), coronary artery disease (CAD), and hyperlipidemia are covered by NHI.

### Ethics statement

The Institutional Review Board at Chi-Mei Medical Center approved this study, which was conducted according to the Declaration of Helsinki. Because the NHIRD data are deidentified, informed consent from the patients was waived. The rights and welfare of the patients were not affected by the waiver.

### Selecting study cohort and comparison cohort

**Fig 1:**
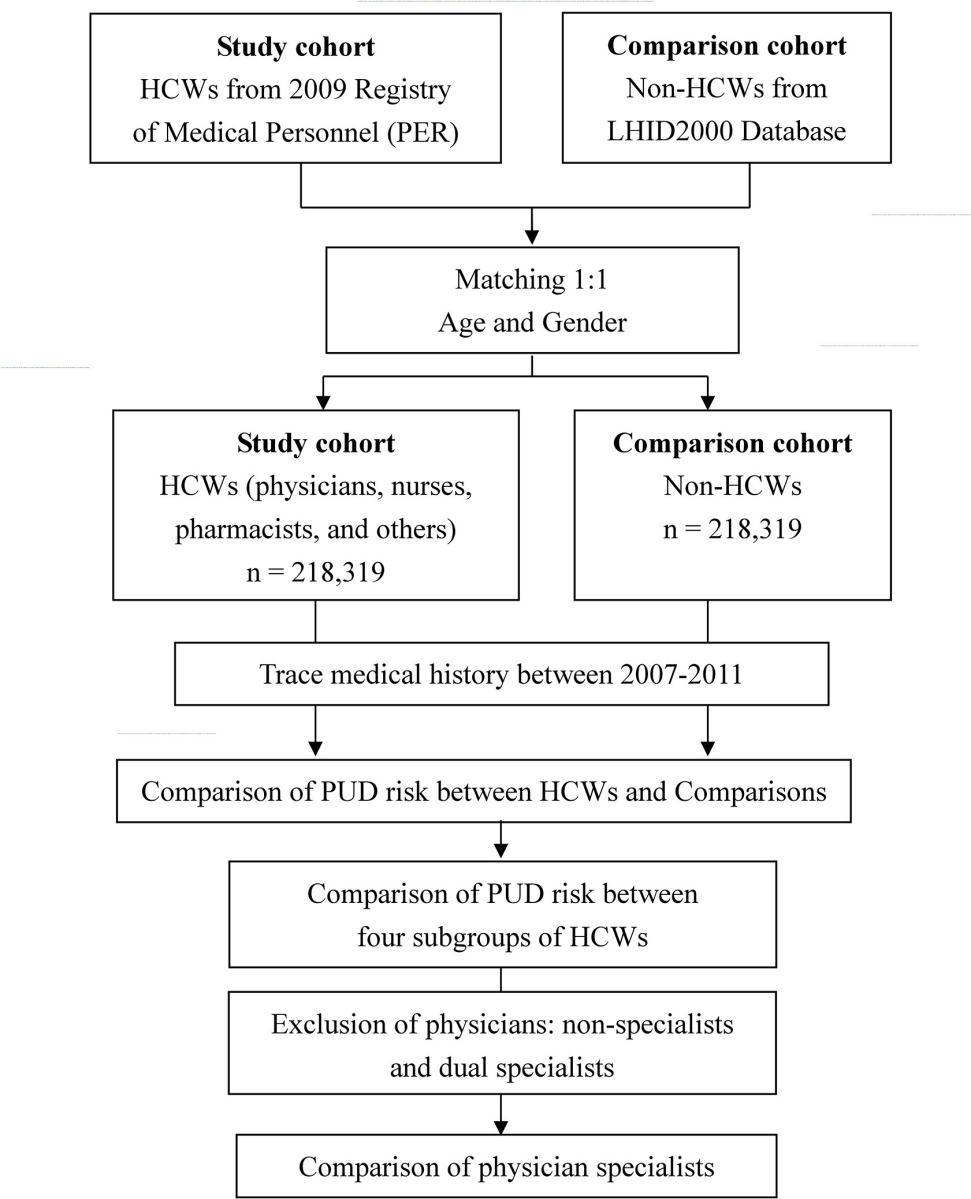
Flow chart for the study. HCWs, health care workers; LHID, Longitudinal Health Insurance Database.

The study cohort (HCWs) were obtained from the Registry of Medical Personnel, which contains records of all registered medical staff in 2009 and all their medical histories between 2007 and 2011 (Fig 1). We classified HCWs into four subgroups for comparison with comparisons: (i) physicians, (ii) nurses, (iii) pharmacists, and (iv) others. Comparisons were non-HCW members of the general population, from the Longitudinal Health Insurance Database 2000 (LHID2000), which contains all claims data of one million (4.34% of the population) beneficiaries who were randomly selected in 2000. The gender, age, claim diagnosis, and healthcare costs between the LHID2000 and all NHI enrollees are not significantly different. Study cohort and comparison cohort were matched 1:1 for age and gender (Fig 1 and Table 1).

**Table 1:** Demographic characteristics and comorbidities for healthcare workers (physicians, nurses, pharmacists, and other HCWs [dieticians, technicians, rehabilitation therapists, and social workers]) and comparisons (general population).

	Physicians	Comparisons		Nurses	Comparisons		Pharmacists	Comparisons		Other HCWs	Comparisons	
	(n = 50,226)	(n = 50,226)	*P*	(n = 122,357)	(n = 122,357)	*P*	(n = 20,677)	(n = 20,677)	*P*	(n = 25,059)	(n = 25,059)	*P*
Age (years)			> 0.999			> 0.999			> 0.999			> 0.999
0–34	12,477 (24.84)	12,477 (24.84)		76,955 (62.89)	76,955 (62.89)		6062 (29.32)	6062 (29.32)		14293 (57.40)	14,293 (57.40)	
35–59	22,001 (43.80)	22,001 (43.80)		38,096 (31.14)	38,096 (31.14)		8358 (40.42)	8358 (40.42)		9025 (36.02)	9025 (36.02)	
≥ 60	15,748 (31.35)	15,748 (31.35)		7306 (5.97)	7306 (5.97)		6257 (30.26)	6257 (30.26)		1741 (6.95)	1741 (6.95)	
Age (years)	44.42 ± 12.16	44.42 ± 12.16	> 0.999	33.55 ± 8.76	33.55 ± 8.76	> 0.999	42.89 ± 11.45	42.89 ± 11.45	> 0.999	34.65 ± 8.78	34.65 ± 8.78	> 0.999
Gender			> 0.999			> 0.999			> 0.999			> 0.999
Male	40,963 (81.56)	40,963 (81.56)		1261 (1.03)	1261 (1.03)		11,376 (55.02)	11,376 (55.02)		8138 (32.48)	8138 (32.48)	
Female	9263 (18.44)	9263 (18.44)	> 0.999	121,096 (98.97)	121,096 (98.97)	> 0.999	9301 (44.98)	9301 (44.98)	> 0.999	16,921 (67.52)	16,921 (67.52)	> 0.999
Comorbidity												
HTN			< 0.0001			0.0671			< 0.0001			0.0004
-Yes	9742 (19.40)	8375 (16.67)		5554 (4.54)	5367 (4.39)		3313 (16.02)	3000 (14.51)		1600 (6.38)	1412 (5.63)	
-No	40,484 (80.60)	41,851 (83.33)		116,803 (95.46)	116,990 (95.61)		17,364 (83.98)	17,677 (85.49)		23,459 (93.62)	23,647 (94.37)	
DM			< 0.0001			0.0004			0.0019			< 0.0001
-Yes	3530 (7.03)	4130 (8.22)		2404 (1.96)	2655 (2.17)		1308 (6.33)	1466 (7.09)		569 (2.27)	708 (2.83)	
-No	46,696 (92.97)	46,096 (91.78)		119,953 (98.04)	119,702 (97.83)		19,369 (93.67)	19,211 (92.91)		24,490 (97.73)	24,351 (97.17)	
CAD			0.0037			0.3671			0.1647			0.3061
-Yes	2709 (5.39)	2505 (4.99)		1180 (0.96)	1224 (1.00)		875 (4.23)	819 (3.96)		340 (1.36)	314 (1.25)	
-No	47,517 (94.61)	47,721 (95.01)		121,177 (99.04)	121,133 (99.00)		19,802 (95.77)	19,858 (96.04)		24,719 (98.64)	24,745 (98.75)	
Hyperlipidemia			< 0.0001			< 0.0001			< 0.0001			< 0.0001
-Yes	8262 (16.45)	5583 (11.12)		6864 (5.61)	4189 (3.42)		2615 (12.65)	2171 (10.50)		1635 (6.52)	1069 (4.27)	
-No	41,964 (83.55)	44,643 (88.88)		115,493 (94.39)	118,168 (96.58)		18,062 (87.35)	18,506 (89.50)		23,424 (93.48)	23,990 (95.73)	
Geographical area			< 0.0001			< 0.0001			< 0.0001			< 0.0001
-North	24,396 (48.57)	26,256 (52.33)		57,346 (46.87)	65,157 (55.75)		9517 (46.03)	11,045 (53.48)		12,060 (48.13)	13,707 (54.74)	
-Central	10,204 (20.32)	8877 (17.69)		22,008 (17.99)	21520 (17.60)		4120 (19.93)	3550 (17.19)		4834 (19.29)	4430 (17.69)	
-South	14,468 (28.81)	14,044 (27.99)		39,447 (32.24)	30,434 (24.90)		6634 (32.08)	5648 (27.35)		7519 (30.01)	6441 (25.72)	
-East	1158 (2.31)	994 (1.98)		3556 (2.91)	2135 (1.75)		406 (1.96)	409 (1.98)		646 (2.58)	460 (1.84)	

HCWs, healthcare workers; DM, diabetes mellitus; HTN, hypertension; CAD, coronary artery disease. Data are n (%) or mean ± standard deviation.

We linked the ICD-9 diagnostic claim codes with the NHI databases. Comorbidities included were HTN (ICD-9 codes 401–405), DM (ICD-9 code 250), CAD (ICD-9 codes 410–414.02), and hyperlipidemia (ICD-9 code 272). These comorbidities were counted if they were diagnosed in ≥ 1 admission care or ≥ 3 ambulatory cares before the January 1, 2009, index medical care date.

### Comparison between HCWs and comparisons

PUD was identified using the ICD-9 code of 531–534. We compared the PUD risk between four subgroups of HCWs and comparisons by tracing their medical histories between 2007 and 2011 (Fig 1). Because this study calculated the prevalence of PUD between 2007 and 2011, therefore, only the diagnosis of PUD made between 2007 and 2011 were included.

### Comparison between HCWs

We compared PUD risk between the four subgroups of HCWs (Fig 1).

### Comparison between physician specialists

Specialists in emergency and critical care might have higher stress and more rotating shift work; therefore, we classified 7 subgroups by physician specialties for comparison: 5 specialties related to emergency and critical care ([i] internal medicine, [ii] surgery, [iii] Ob/Gyn [obstetrics/gynecology], [iv] pediatrics, and [v] emergency medicine), family medicine, and others (Fig 1). Physicians with dual specialties were excluded because it was difficult to assign them to individual specialties. Residents were also excluded because of their short practice time in individual specialties.

### Statistical analyses

Student’s *t* test was used for continuous variables and Pearson χ^2^ tests for categorical variables when comparing demographic characteristics and comorbidities between HCWs and comparisons. Conditional logistic regression was used to compare the risk for PUD between HCWs and comparisons. Unconditional logistic regression was used to compare the HCWs and to compare the physician specialists. SAS 9.3.1 for Windows (SAS Institute, Cary, NC, USA) was used for all analyses. Significance was set at *P* < 0.05 (two-tailed).

## Results

### Demographic characteristics and comorbidities of study cohort (HCWs) and comparison cohort (general population)

In total, 50,226 physicians, 122,357 nurses, 20,677 pharmacists, and 25,059 other HCWs, and an identical number of age- and gender-matched comparisons were recruited (Table 1). The median age of the physicians was 44.42 ± 12.16 years, of the nurses was 33.55 ± 8.76 years, of the pharmacists was 42.89 ± 11.45 years, and of the other HCWs was 34.65 ± 8.78 years (Table 1). The percentages of female physicians, nurses, pharmacists, and other HCWs were 18.44%, 98.97%, 44.98%, and 67.52%, respectively. All four subgroups of HCWs had a lower prevalence of DM than did the comparisons. Physicians, pharmacists, and other HCWs had a higher prevalence of HTN than did the comparisons, but nurses did not. Physicians had a higher prevalence of CAD than did the comparisons but others did not. All four subgroups of HCWs had a higher prevalence of hyperlipidemia than did the comparisons.

### Comparison of PUD risk between HCWs and comparisons

The prevalences of PUD in physicians, nurses, pharmacists, and other HCWs were 10.28%, 9.53%, 9.05%, and 9.10%, respectively (Table 2). Compared with comparisons, nurses and other HCWs had a significantly higher risk for PUD than did comparisons (odds ratio [OR]: 1.477, 95% confidence interval [CI]: 1.433–1.521 and OR: 1.328, 95% CI: 1.245–1.418, respectively). Pharmacists had a significantly lower risk for PUD than did comparisons (OR: 0.884, 95% CI: 0.828–0.945). The risk for PUD between physicians and comparisons was not significantly different (OR: 1.029, 95% CI: 0.987–1.072).

**Table 2:** Comparison of risk for PUD between physicians, pharmacists, nurses, and other HCWs with comparisons (conditional logistical regression analysis).

	Number (%)	OR (95% CI)
Physicians (n = 50,226)	5,162 (10.28)	1.029 (0.987–1.072)
Comparisons (n = 50,226)	5,035 (10.02)	1.00
Nurses (n = 122,357)	11,658 (9.53)	1.477 (1.433–1.521)**
Comparisons (n = 122,357)	8,193 (6.70)	1.00
Pharmacists (n = 20,677)	1,871 (9.05)	0.884 (0.828–0.945)**
Comparisons (n = 20,677)	2,087 (10.09)	1.00
Other HCWs (n = 25,059)	2,281 (9.10)	1.328 (1.245–1.418)**
Comparisons (n = 25,059)	1,760 (7.02)	1.00

HCWs, healthcare workers; PUD, peptic ulcer disease; OR, odds ratio; CI, confidence interval; DM, diabetes mellitus; HTN, hypertension; CAD, coronary artery disease.

**P < 0.001.

### The risk for PUD between the four subgroups of HCWs

When we used the other HCWs as the reference, physicians and nurses had a significantly higher risk for PUD (OR: 1.144, 95% CI: 1.086–1.205 and OR: 1.052, 95% CI: 1.003–1.102, respectively), but pharmacists did not (Table 3).

**Table 3:** Comparison of risk for PUD between four subgroups of HCWs (unconditional logistic regression).

	Number (%)	OR (95% CI)
Physicians (n = 50,226)	5,162 (10.28)	1.144 (1.086–1.205)**
Nurses (n = 122,357)	11,658 (4.76)	1.052 (1.003–1.102)*
Pharmacists (n = 20,677)	1,871 (9.05)	0.993 (0.932–1.059)
Other HCWs (n = 25,059)	2,281 (9.10)	1.00

HCWs, healthcare workers; PUD, peptic ulcer disease; OR, odds ratio; CI, confidence interval; DM, diabetes mellitus; HTN, hypertension; CAD, coronary artery disease.

*P < 0.05.

**P < 0.001.

### The risk for PUD between the physician specialties

Physicians specialized in internal medicine, surgery, Ob/Gyn, and family medicine had a significantly higher risk for PUD than did physicians in other specialties (Table 4). However, emergency medicine physicians had a significantly lower risk for PUD than did other specialists (OR: 0.544, 95% CI: 0.359–0.822).

**Table 4:** Comparison of risk for PUD between physician specialties (unconditional logistic regression).

	Number (%)	OR (95% CI)
Specialists		
Internal medicine (n = 6,110)	812 (13.29)	1.579 (1.441–1.731)**
Surgery (n = 4,095)	590 (14.41)	1.734 (1.565–1.922)**
Ob/Gyn (n = 1,978)	227 (11.48)	1.336 (1.151–1.550)**
Pediatrics (n = 2,774)	231 (8.33)	0.936 (0.809–1.082)
Emergency medicine (n = 479)	24 (5.01)	0.544 (0.359–0.822)*
Family medicine (n = 2,568)	348 (13.55)	1.615 (1.425–1.831)**
Other specialties (n = 15,995)	1,415 (8.85)	1.00

PUD, peptic ulcer disease; OR, odds ratio; CI, confidence interval; DM, diabetes mellitus; HTN, hypertension; CAD, coronary artery disease.

*P < 0.05.

**P < 0.001.

## Discussion

This is the first study to delineate the risk for PUD in HCWs compared with general population. Nurses and other HCWs had a higher risk for PUD than did the general population, but pharmacists had a lower risk. Within the four subgroups of HCWs, physicians and nurses had the highest risk and pharmacists had the lowest. Within the physician specialties subgroups, internal medicine, surgery, Ob/Gyn, and family medicine specialists had higher risks than did other specialists, emergency medicine specialists had the lowest. These findings may help us make better health policy in the future.

Higher job stress and more frequent rotating shift work might be the reason that physicians and nurses had the highest risk for PUD. A recent study in Taiwan [9] showed that the prevalences of high work-related burnout was 66% in nurses, 61.8% in physician assistants, 38.6% in physicians, 36.1% in administrative staff, and 31.9% in medical technicians. Although PUD cannot be regarded as an occupational disease, it has a higher incidence among professional people and those working under stress [2].

Many epidemiological studies [7] have reported that PUD is related to rotating shift work. Nurses have the highest percentage of shift work (74%) in Taiwan: physicians (46%), medical technicians (38%), physician assistants (29.4%), and administrators (21.2%) [9]. The gastrointestinal system has a circadian rhythm that has been demonstrated for bowel movement, secretion of gastric juices, bile acid synthesis, and immune activity; therefore, shift work might disrupt the normal circadian rhythm and cause increased risk for PUD [7]. Other studies have shown that *Helicobacter pylori* infection was more prevalent in shift workers than in day workers (46.1% versus 34.6%) [10], and that *Helicobacter pylori*-infected shift workers have a higher prevalence of peptic ulcer than do infected day workers (28.7% versus 9.3%) [11]. That shift work leads to the deterioration of the natural defense to *Helicobacter pylori* infection might explain these findings.

Job stress and shift work might also explain why physicians specialized in internal medicine, surgery, and Ob/Gyn had a higher risk for PUD than did other specialists. All of internists, surgeons, and gynecologists always need to manage emergency and critical patients, which might cause higher stress, workload, and longer working time, including on-call and shift work. A study in Taiwan [12] showed that physician specialties that involve emergency and critical care, working ≥ 8 h/shift, serving ≥ 51 patients per shift, and being on call ≥ 41 times per week were strongly associated with a high level of burnout, and a study in the USA [13] showed that 32% of actively practicing surgeons showed high levels of emotional exhaustion and 13% showed high levels of depersonalization. A study in the UK [14] also showed that 32% of surgeons had high burnout, psychiatric morbidity, and work dissatisfaction, all of which were likely to adversely affect their health, their patients’ satisfaction, and the quality of the service they provided.

Family physicians had aspects of stress different from those of internists, surgeons, and gynecologists that were risk factors for developing PUD. A study in Canada [15] showed that almost half of family physicians had high levels of emotional exhaustion and depersonalization. The sources of stress were too much paperwork, feeling undervalued, feeling unsupported, long waits for specialists and tests, and having to abide by rules and regulations. An international study in Europe [16] also revealed that 43% of the responding family physicians scored high for emotional exhaustion burnout, 35% for depersonalization, and 32% for personal accomplishment.

The lower average working time and no on-call stress might explain why emergency physicians had a lower risk for PUD. A study about acute myocardial infarction (AMI) [17] in physicians showed that emergency physicians had equal AMI risk with other specialties. Others [18] have reported that although emergency physicians experience high level of work stress, their psychological health was greatly improved by coping strategies and social support. In Taiwan, emergency physicians work an average of 45 h/week, lower than the average of all physicians (49.5% > 57 h/week), and the longer the working time, the higher the level of stress is [12]. In addition, emergency physicians have no duty after getting off work, which may help them cope with and relieve the stress they feel during work.

This study has some limitations. First, we did not match or adjust for some risk factors for PUD because of the unavailability of reliable data or no data at all. In particular, *Helicobacter pylori* infection and NSAIDs use are two well established risk factors that we did not account for and an imbalance in the distributions of these two factors among the comparison groups may lead to confounding. Likewise, we did not account for other risk factors such as BMI, lifestyle, or other environmental factors. Further studies that can account for the effects of these factors are warranted. Second, we did not have direct measurements of the stress such as heavy workload, long working hour, workplace stress, rotating shift work, and coping skills even though the argument of a role of stress is biologically plausible. Third, our 5-year follow-up might not be long enough to confirm our findings.Forth, we did not analyze subgroups of nurses, pharmacists, or other HCWs; therefore, the risks for PUD in these different HCW specialties are unknown. More studies are needed to clarify these risks. Finally, our findings might not be generalizable to the HCWs in other countries.

## Conclusions

This is the first study to reveal the risk for PUD in physicians, nurses, pharmacists, and other HCWs. Nurses and other HCWs had a significantly higher risk than did the general population. Physicians and nurses had a higher PUD risk than other HCWs. Physicians specialized in internal medicine, surgery, Ob/Gyn, and family medicine had higher risks than did those in other specialties; however, emergency physicians had a lower risk. The possible explanations for the increased risk for PUD may be working hours, job stress, shift work, and coping skills. Our findings provide epidemiological evidence for health policy but additional study is needed to clarify the mechanisms.
